# Evaluation of Sociodemographic Factors among Diabetic Patients with Urinary Tract Infections in Kisii Referral Hospital, Kenya

**DOI:** 10.1155/2020/5053867

**Published:** 2020-07-07

**Authors:** Vincent M. Mageto, Oliver W. Mbuthia, Caroline J. Ngetsa, Dinah O. Moraa, Erick O. Okoyo, Scholastica G. Mathenge, Wachuka G. Njoroge

**Affiliations:** ^1^International Rescue Committee, Nairobi, Kenya; ^2^Department of Medical Microbiology, University of Nairobi, Nairobi, Kenya; ^3^Department of Bioscience, KEMRI‐Wellcome Trust Research Programme, Kilifi, Kenya; ^4^Department of Medical Laboratory Sciences, Kenya Methodist University, Meru, Kenya; ^5^Department of Health Sciences, Eldoret National Polytechnic, Eldoret, Kenya; ^6^Department of Medical Laboratory Sciences, Kenyatta University, Nairobi, Kenya

## Abstract

People with noninsulin-dependent diabetes mellitus are prone to urinary tract infections. There is a wide gap of information in developing countries regarding the sociodemographic factors linked to UTI among diabetics and the gender disparity among the same. Sociodemographic factors differ with geographical location and many other factors, and this makes them an important aspect that can influence the social burden of UTI among diabetics. The objective of this study was to determine the association between sociodemographic factors and UTI among diabetics. The study was carried out in the Kisii Teaching and Referral Hospital in Kenya. One hundred and eighty diabetic patients were enrolled in cross-sectional study design. Clean-catch midstream urine was collected from all participants and cultured in cysteine lactose electrolyte deficient agar for bacterial isolation. Classification of a positive culture for urinary tract infection was based on more than 100,000 (≥10^5^) colony-forming units of a single bacterial species. The data were analyzed using frequencies, chi-square (*p* < 0.05), and logic regression with the help of the Statistical Package for the Social Sciences (SPSS) version 20 to find the odds ratio. One hundred and seven participants were male (59.4%), and 73 (40.6%) were female. The majority of the participants were between the age of 55 and 59 years old (77.2%), and 125 participants (69.4%) had attained tertiary education as the highest level of education. The overall prevalence of urinary tract infections was 20.6% with 37 participants testing positive for urinary tract infection. Age was found to have a significant association with urinary tract infection (*p*=0.002) while gender (*p*=0.45) and level of education (*p*=0.11) showed no significant association with urinary tract infections among diabetic patients. These findings suggest that age was the biggest association factor that influenced urinary tract infections among diabetic patients.

## 1. Introduction

Sociodemographic factors are in close proximity to the behaviors that humans develop, and this makes them have a significant influence on the infections people acquire. Due to the diverse distribution of sociodemographic factors based on geographical location, the occurrence of infections depends on these factors. In this study, the association of sociodemographic factors is being assessed with the occurrence of urinary tract infections among diabetic patients.

Urinary tract infections are ranked among the most common infections affecting people of all ages. Globally, it is estimated that 150 million people contract urinary tract infections annually [1]. This shows the huge burden that health systems in the world struggle within the management of urinary tract infections. Coinfection of urinary tract infections and diabetes means that the burden of management is even heavier and this reveals the severity of the problem. Due to the impairment of the immune system because of decreased cellular responses, urinary tract infections (UTIs) are commonly found in diabetic patients [1]. Poor metabolic control has also been found to be another reason why patients with diabetes are prone to UTI [1, 2]. Dysfunctional voiding and retention of urine in the bladder reduces the number of bacteria that are physically eliminated from the urinary tract through micturition [3]. In premenopausal women, lactobacilli make up to 90% of the normal flora in the vagina [4]. Decreased numbers of vaginal lactobacilli predispose women to colonization with pathogens responsible for UTI [5].

When the host defense mechanisms become overwhelmed by pathogens, an infection occurs [6]. There is a wide range of bacteria that can infect the urinary tract, leading to UTI. According to Walsh and Collyns [7], there are factors that may facilitate the progression of infection faster than others may. These factors may be associated with the host or the pathogen. The host factors that predispose an individual to UTI include gender [8]. It is projected that up to half of women will have at least one episode of UTI in their lifetime. Women have a shorter urethra making it easier for bacteria to travel up to the bladder and cause an infection there [9]. The female anatomy also has the urethra closer to the anus, consequently making it easier for bacteria from the rectum to find their way to the urethra [10].

## 2. Materials and Methods

### 2.1. Study Site

The study was carried out in the Kisii Teaching and Referral Hospital in Kenya during the period of July–December 2017. Patients who have not been on antibiotic therapy for at least two weeks were eligible for the study. Diabetes patients were sampled from the diabetic outpatient clinic. Every fourth patient visiting the clinic was selected to take part in the study.

### 2.2. Sample Collection

All participants signed consent forms prior to the sample collection. A total of 180 urine samples were collected from the participants using the clean-catch midstream morning technique in sterile containers. The procedure was well explained to the participants so as to ensure that there was no contamination in the urine samples collected. Each and every participant was asked to fill in a pretested questionnaire before a urine sample was collected. The questionnaire was used as a tool for collecting sociodemographic information and some clinical information of the participant. As soon as the samples were collected, they were labeled and transported to the laboratory for analysis in a cool box. About 20 ml of urine was collected from every participant. All urine samples were analyzed in the Kisii Teaching and Referral Hospital microbiology laboratory.

### 2.3. Sample Processing

With the help of a standard quantitative wire loop, all samples were inoculated onto cysteine lactose electrolyte deficient (CLED) agar. The streaking technique was used in inoculating the urine samples in the culture media. The plates were incubated at 37 degrees Celsius for 24 hours. The resultant growth was classified as significant or nonsignificant. Nonsignificant samples were discarded in accordance with proper biological waste management protocol. In line with the WHO definition of UTI, culture plates showing ≥10^5^ colony-forming units per mL of a single bacterial species were considered significant [11]. Bacteriuria in participants who presented with symptoms of UTI was symptomatic bacteriuria while in those who did not present with symptoms of UTI, it was asymptomatic bacteriuria.

### 2.4. Data Analysis

All the data were entered into the computer using the Statistical Package for the Social Sciences software for Windows version 20 (SPSS Inc., Chicago, IL, USA) and checked before analysis was done. Frequencies and proportions of the sociodemographic factors were worked out and compared against the culture-positive group using linear regression and chi-square tests, respectively. The culture-positive cases were used as dependent variables, and sociodemographic cases were used as independent variables. Probability values of <0.05 were considered statistically significant for all results.

## 3. Results

Results from Table 1 reveal that among the participants who took part in the study, 107 were male, representing 59.4% of the total participants, while a minority of 73 participants were female, representing 40.6% of the total participants. The age group between 55 and 59 years old had the highest proportion of participants (139) representing 77.2%, followed by the group between 60 and 64 years old with 26 participants representing 14.4%, then the group between 50 and 54 years old with 11 participants representing 6.1%, and finally, the age group between 65 and 69 years old had the least proportion of 4 participants representing 2.2% of the total number of participants. Considering the level of education of the participants, the majority of them had attained their highest level of education from a tertiary institution at 69.4% (125 participants), followed by secondary school at 21.7% (39 participants), and finally, the primary school at 8.9% (16 participants). The results are shown in Table 1.

**Figure 1:**
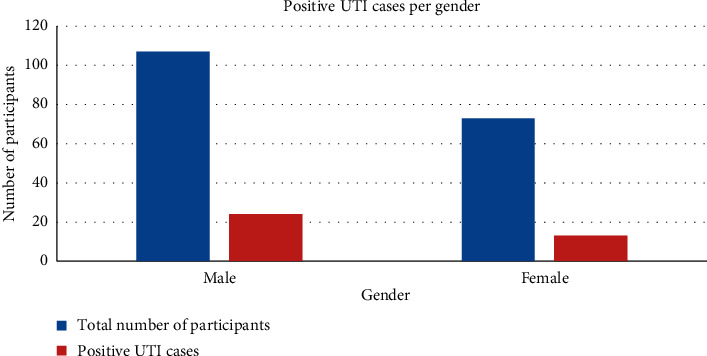
Total number of participants and positive UTI cases per gender.

The gender of participants exhibited no significant difference in reference to urinary tract infection (chi-square (*χ*^2^) = 0.568; degree of freedom (d*f*) = 1; *p* value = 0.45). As shown in Figure 1, among the 37 participants who tested positive for urinary tract infections, 64.9% (24) were male, while 35.1% (13) were female. Among the male participants, 22.4% (24) tested positive for UTI compared to 17.8% (13) of the female participants.

**Figure 2:**
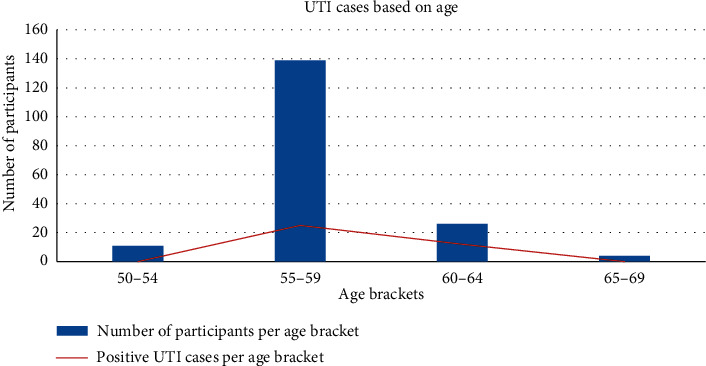
Total number of participants and positive UTI cases per age bracket.

The age of the participants revealed significant difference in urinary tract infections (*χ*^2^ = 14.876; d*f* = 3; *p* = 0.002). As shown in Figure 2, no participants between the ages of 50 and 55 years old tested positive for UTI. Eighteen percent (25) of the participants between the ages of 55 and 59 years old tested positive for UTI, while 46.2% (12) of participants between 60 and 64 years old tested positive for UTI. All the participants (2) between the ages of 65 and 69 years tested negative for urinary tract infections.

**Figure 3:**
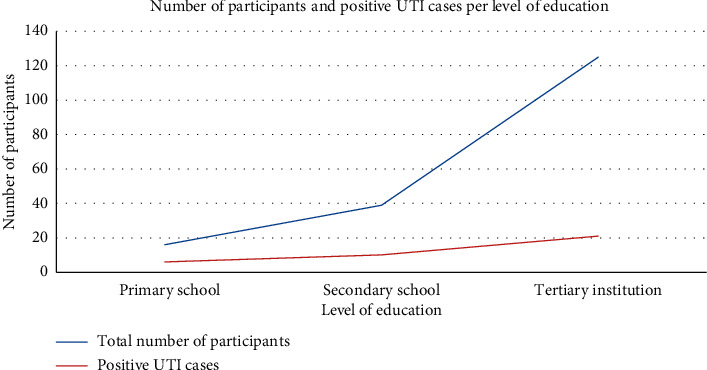
Total number of participants and positive UTI cases per level of education attained.

The level of education showed no significant difference in reference to urinary tract infections among participants (*χ*^2^ = 4.51; d*f* = 2; *p* = 0.11). As shown in Figure 3, among the participants whose highest level of education was primary school, 37.5% (6) tested positive while 62.5% (10) tested negative for urinary tract infections. Among participants whose highest level of education was secondary school, ten of them (25.6%) tested positive while 29 from the same category (74.4%) tested negative for urinary tract infections. Among participants whose highest level of education was tertiary institutions, 16.8% (21) tested positive while 83.2% (104) tested negative for urinary tract infections.

## 4. Discussion

The male participants were 1.335 (odds ratio) times more likely to test positive for UTI than female participants. These findings are in agreement with those from a study carried out in Sudan where more males tested positive for UTI than females at 22.3% and 15.1%, respectively [8]. Another study carried out in the United Arab Emirates revealed contrasting findings with more females testing positive for UTI at 37.0% compared to males at 34.0% [12]. Due to varying behavioral practices among participants in different geographical locations, it is possible that this may influence the different prevalence of UTI between genders.

There was a significant difference between age and UTI (*p* = 0.004). As the age of participants increased by five years, the probability of a participant to test positive for UTI increased by 1.324 (odds ratio) times. Similar findings have been obtained in studies in the USA [13] and Canada [14] where it was revealed that UTI cases increased with age. Contrasting findings were obtained in Sudan where age did not have a significant association with urinary tract infections [9]. The reason why most studies revealed a significant association with UTI is probably due to the deteriorating state of the immunity that comes as a result of the diabetic condition of the participant.

Compared to the respective total number of participants based on the level of education, the majority of the participants that tested positive for UTI had their highest level of education from primary school (37.5%), followed by secondary school (25.6%), and lastly, tertiary institutions (16.8%). Findings from studies in Sudan [8] and Israel [15] have revealed similar findings of no significant association between the level of education and urinary tract infection.

Urinary tract infections are classified as upper and lower and as complicated or uncomplicated [1]. The coinfection of UTI in diabetic patients is an increasing cause of morbidity across the globe. As a result of limited resources to curb the infections before they become severe, low-income economy nations suffer the greatest burden [16]. The occurrence of urinary tract infection can exhibit symptoms or not show any symptoms. Findings from a study in Ethiopia showed 13.6% of UTI among diabetics to be symptomatic while 10.4% showed no symptoms [17]. Contrary to these findings, more asymptomatic than symptomatic UTI cases were revealed in Sudan. Out of 200 diabetic patients enrolled for the study, 20.9% of UTI cases among diabetics were found to be asymptomatic while 17.1% were symptomatic [18].

Regarding sociodemographic characteristics relating to UTI and diabetes, there are contradicting findings that have been reported in various studies. In Sudan, study findings revealed that there was no association between age and UTI among diabetics [8]. Another study carried out in Sweden revealed a significant association between age and UTI among diabetics [9]. However, studies strongly support the significant association of UTI among diabetic patients. Findings from a study in Holland revealed that among diabetic women, 7.1% had relapsing UTI while 2.0% of nondiabetic women had relapsing UTI [19]. This is a strong supporter of the fact that diabetic patients are at a higher risk of contracting urinary tract infections than nondiabetic individuals.

## Figures and Tables

**Table 1:** Sociodemographic factors associated with UTI among noninsulin-dependent diabetes mellitus.

Variables	Frequency	(%)	Positive UTI cases	(%)
*Gender*				
Male	107	(59.4)	24	(64.9)
Female	73	(40.6)	13	(35.1)

*Age in years*				
50–54	11	(6.1)	0	(0)
55–59	139	(77.2)	25	(18)
60–64	26	(14.4)	12	(46.2)
65–69	4	(2.2)	0	(0)

*Highest level of education*				
Primary school	16	(8.9)	6	(37.5)
Secondary school	39	(21.7)	10	(25.6)
Tertiary institution	125	(69.4)	21	(16.8)

## Data Availability

The data supporting the findings in this study are available from the corresponding author upon request.
